# Nanoporous TiN/TiO_2_/Alumina Membrane for Photoelectrochemical Hydrogen Production from Sewage Water

**DOI:** 10.3390/nano11102617

**Published:** 2021-10-06

**Authors:** Abdullah Almohammedi, Mohamed Shaban, Huda Mostafa, Mohamed Rabia

**Affiliations:** 1 Department of Physics, Faculty of Science, Islamic University in Madinah, Al-Madinah Al-Munawarah 42351, Saudi Arabia; ard.almohammedi@hotmail.com; 2 Nanophotonics and Applications (NPA) Lab, Physics Department, Faculty of Science, Beni-Suef University, Beni-Suef 62514, Egypt; hudamostafa55@gmail.com (H.M.); mohamedchem@science.bsu.edu.eg (M.R.); 3 Polymer Research Laboratory, Chemistry Department, Faculty of Science, Beni-Suef University, Beni-Suef 62511, Egypt

**Keywords:** Al_2_O_3_ template, nanomaterials, surface plasmon resonance, photoelectrochemical hydrogen production, sewage water, solar energy conversion

## Abstract

An aluminum oxide, Al_2_O_3_, template is prepared using a novel Ni imprinting method with high hexagonal pore accuracy and order. The pore diameter after the widening process is about 320 nm. TiO_2_ layer is deposited inside the template using atomic layer deposition (ALD) followed by the deposition of 6 nm TiN thin film over the TiO_2_ using a direct current (DC) sputtering unit. The prepared nanotubular TiN/TiO_2_/Al_2_O_3_ was fully characterized using different analytical tools such as X-ray diffraction (XRD), Energy-dispersive X-ray (EDX) spectroscopy, scanning electron microscopy (SEM), and optical UV-Vis spectroscopy. Exploring the current-voltage relationships under different light intensities, wavelengths, and temperatures was used to investigate the electrode’s application before and after Au coating for H_2_ production from sewage water splitting without the use of any sacrificing agents. All thermodynamic parameters were determined, as well as quantum efficiency (QE) and incident photon to current conversion efficiency (IPCE). The QE was 0.25% and 0.34% at 400 mW·cm^−2^ for the photoelectrode before and after Au coating, respectively. Also, the activation energy was 27.22 and 18.84 kJ·mol^−1^, the enthalpy was 24.26 and 15.77 J·mol^−1^, and the entropy was 238.1 and 211.5 kJ^−1^·mol^−1^ before and after Au coating, respectively. Because of its high stability and low cost, the prepared photoelectrode may be suitable for industrial applications.

## 1. Introduction

Renewable energy sources are very important for our life, whereas most of these sources are based on photocatalytic materials that are used in the preparation of solar cells, photodetectors, and photocatalytic electrodes [1,2,3]. The production of H_2_ gas due to water-splitting reaction is a very important energy source for airplanes and factories that used H_2_ fuel [4]. The prepared photoelectrode must have high efficiency for water splitting and properties that qualify it for industrial applications, such as low cost, easy preparation, and easy operation [5,6]. The working of the electrode can be carried out under direct sunlight or using another artificial light. Many semiconductor materials were used for the synthesis of the electrode, including oxides, sulfides, and some polymers [7,8]. The properties of the photocatalytic materials improve with increasing the active sites inside the materials, in which the nanowires or nanotube morphology increase the active sites through increasing the surface area of the materials [9,10]. The other way for increasing the efficiency of the electrode is using plasmonic materials for light capture, in which a surface plasmonic resonance is produced from the oscillation of electrons in the conductive bandgap [11].

The most used plasmonic materials are noble metals, such as Au, Ru, Rh, and Pt, but these metals are very expensive [12]. Rabia et al. used Au to enhance the water-splitting reaction for H_2_ production under light [13]. Shi et al. used Pt to enhance the activity of CdS materials for water splitting, in which the photoactivity is enhanced very much by using Pt metal [14]. At the same time, many researchers used active metals such as Co, Fe, Ni, and Cu as plasmonic materials. This appears in Cu/ZnO materials, in which the Cu nanomaterials enhance the light capture and efficiency for the light detection in the prepared photodetector [15]. Although these elements are cheap, they are active and easy to form oxides.

With its high noble features and coating protection, metal nitride ceramic can serve as a photo-plasmonic resonance material for light capture, comparable to noble plasmonic metal. These behaviors originate from the chemical stability and high melting point (>2000 K) [16]. Moreover, these metal nitrides are used in designing devices with spectral windows and operating conditions more efficiently in hazardous environmental conditions than noble metals [17]. Some studies were carried out for comparing the effect of TiN and ZrN plasmonic materials on the sensing properties of a plasmonic refractometry sensor with noble metals, finding that the nitride metals were more efficient than the noble metals [16,18,19,20].

Due to the good properties of the TiN material, such as high hardness, nontoxicity, high thermal conductivity, high corrosion resistance, chemical stability, and high UV-Vis light absorbance, Mohamed et al. used a 9 nm TiN layer to enhance the properties of TiO_2_ photoactive material for solar cells [18]. With an increase in TiN thickness to 9 nm, the optical properties, absorption, and bandgap improved, resulting in increased photocurrent. Awad et al. used tri-layered TiO_2_/TiN/TiO_2_ self-cleaning systems of different TiN thicknesses to optimize their optical properties and facilitate the photodegradation of the organic materials [21]. Also, many researchers have concentrated on the properties of TiN and TiN-based nanostructures for application in many devices, such as fuel cells, supercapacitors, superconductors, and solar cells [22,23,24]. 

Photocatalytic properties of TiO_2_ materials, especially in structures with a large surface area, such as nanowires and nanorods, are of particular interest [25]. TiO_2_ nanotubes tolerate more active sites on their internal and external surfaces that increase their activity in light absorption, in addition to their large surface area-to-volume ratios. TiO_2_ also has several desirable characteristics, including nontoxicity, low cost, ease of preparation, and high stability [22]. These properties qualify TiO_2_ materials for application in sensors, supercapacitor, H_2_ production, solar cell, and light absorbance [26]. Since TiO_2_ has a wide bandgap > 3 eV, it can only be used in photocatalytic and light detection devices that operate in the UV range [27]. 

Many studies on H_2_ generation by water splitting have recently been released, but almost all of them use a sacrificing agent, such as NaOH, HCl, Na_2_S_2_O_3_, or Na_2_SO_3_ for water splitting [28,29,30,31]. Also, the majority of these studies do not demonstrate long-term H_2_ production stability. Furthermore, the morphology of the microsized materials is uncontrolled [30,31,32]. In this study, a TiN/TiO_2_/Al_2_O_3_ template photoelectrode was created and used to generate H_2_ from sewage water splitting without the use of any sacrificing agents. The effect of depositing a thin Au film on the TiN/TiO_2_/Al_2_O_3_ electrode was also investigated. An Al_2_O_3_ template was prepared with high hexagonal ordering using the Ni imprinting technique. The TiO_2_ was deposited using the ALD technique, whereas the thin TiN layer was deposited using the DC sputtering technique. The electrode’s application for H_2_ production was studied by exploration the current–voltage relationship under various light intensities, wavelengths, and temperatures. The quantum efficiency (QE), incident photon to current conversion efficiency (IPCE), and all thermodynamic parameters were calculated. The prepared photoelectrode can be applicable in the industrial field with high stability and low cost.

## 2. Experimental Methods

### 2.1. Al_2_O_3_ Template Synthesis

The synthesis of the Al_2_O_3_ template was carried out with a novel method of high controllability in the shape and size of the pores. The aim is to prepare pores with a hexagonal shape with a pore diameter of about 320 nm. For the preparation of the Al_2_O_3_ template, Al metal (99.99%) was used, in which an electropolishing process was carried out for removing any impurities or corrosions. To determine the position of the pores, the Ni imprinting method was applied at a pressure of ~10 kN/cm^2^ for 180 s using an oil pump [24]. Then, a two-step anodization process is carried out using a mixture of H_3_PO_4_, ethylene glycol, and H_2_O with a volume ratio of 1:100:200. The first step was carried out for 15 min, whereas the second step was performed for 120 min at 2 °C. The first step was etched using a mixture of H_2_CrO_4_ and H_3_PO_4_ with concentrations of 1.5 wt% and 6 wt%, respectively. As a final step, the nanopores were widened at 60 °C for 20 min.

### 2.2. Synthesis of TiN/TiO_2_ and Au/TiN/TiO_2_

TiN/TiO_2_ nanotubes were prepared inside the Al_2_O_3_ template through two different steps. The pore of the template was about 320 nm with a length of about 1 µm. First, TiO_2_ was synthesized by the atomic layer deposition device (ALD, Picosun SUNALE R150 ALD, Poscosun, Espoo, Finland) at 300 °C for 300 running cycles with a rate of 0.3 nm/s. TiO_2_ is prepared from TiCl_4_ as Ti source and H_2_O as O source. After that, TiN thin film (8 nm) was deposited over TiO_2_ using DC sputtering device under initial and working pressures of 6.7 × 10^−7^ and 1.3 × 10^−2^ mbar, respectively. The sputtering process was carried out from a mixture of N_2_ and Ar gases with volumes of 75 and 25 sccm, respectively. The Ti source was a Ti-metal (99.99%). Finally, for synthesis Au/TiN/TiO_2_, Au nanoparticles are sputter-coated over TiN/TiO_2_/Al_2_O_3_ template under vacuum conditions for 3 min. Au deposition was carried out utilizing a fairly basic sputter-coater at pressure 2 torr (low vacuum), current 13 mA, and a distance of 8 cm in front of the Au target. The rate of growth of the Au layer was 5 nm/min. 

### 2.3. Samples Characterization 

The prepared template and nanotubes were characterized using different devices. The chemical structure was confirmed using an X-ray diffractometer (XRD, Bruker/Siemens D5000, Aubrey, TX, USA) by Kα radiation of Cu (λ = 1.5405 Å) at 30 mA, and 40 kV. Moreover, the elements inside the materials and nanomorphologies were determined using a scanning electron microscope (SEM, Auriga Zeiss FIB, Zeiss company, Oberkochen, Germany) provided with energy-dispersive X-ray (EDX, Zeiss company, Oberkochen, Germany) unit. The optical properties were characterized using a double beam spectrophotometer (Perkin Elmer Lambda 950, Perkin Elmener company, Waltham, MA 02451, USA).

### 2.4. Water-Splitting Test

The water-splitting test reaction was carried out under a solar simulator from a two-electrode cell, in which the TiN/TiO_2_/Al_2_O_3_ or Au/TiN/TiO_2_/Al_2_O_3_ represented the working electrode that worked as a cathode, whereas a graphite electrode with the same dimension was worked as an anode, Figure S1 (Supplementary Data). The chemical composition of the sewerage water is mentioned in Table S1. The pH value was 5 for this wastewater. The effects of light intensity (100 to 400 mW·cm^−2^), incident wavelength (405 to 636 nm), temperature reaction (30 to 70 °C), and time stability were studied for water splitting for H_2_ and O_2_ evolutions. The effect of monochromatic light on the water splitting is tested using optical filters of different wavelengths; 405, 470, 490, 508, and 636 nm.

## 3. Results and Discussion

### 3.1. SEM and XRD Studies

**Figure 1:**
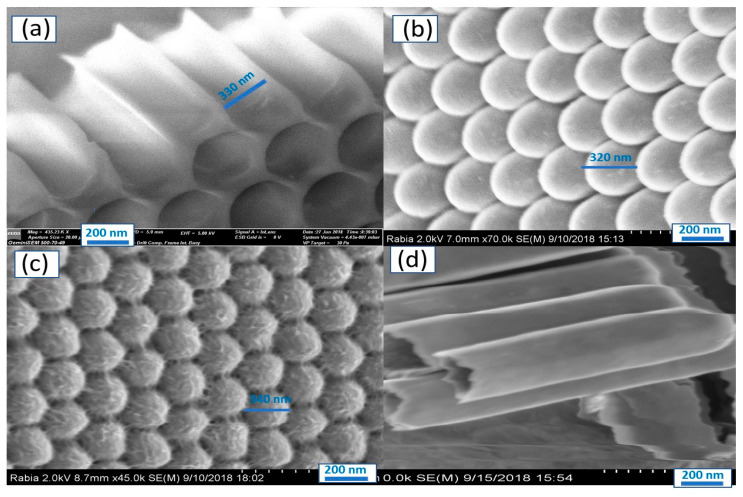
SEM images of (**a**) Al_2_O_3_ template after pore widening, (**b**) TiO_2_, (**c**) TiN/TiO_2_, and (**d**) TiN/TiO_2_ a cross-section.

**Figure 2:**
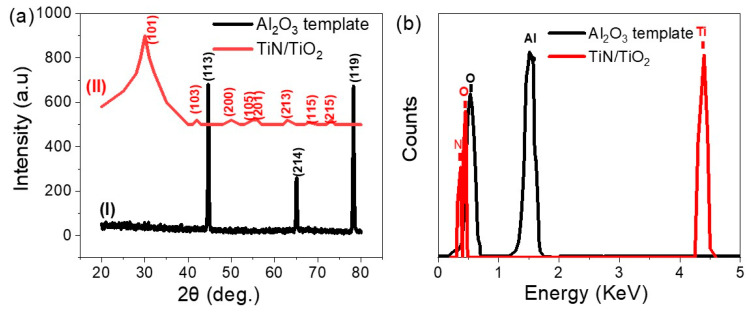
(**a**) XRD and (**b**) EDX of Al_2_O_3_ template and TiN/TiO_2_.

The morphological analysis of the Al_2_O_3_ template after pore widening at 60 °C is mentioned in Figure 1a. From this figure, the Al_2_O_3_ template is prepared with high accuracy and ordering with a hexagonal shape, in which the pore diameter is about 330 nm. Figure 2b displayed the bottom of the membrane after the deposition of TiO_2_ by the ALD. The bottoms of the tubes are uniformly coated, whereas the outer diameter of the bottom is about 320 nm. Moreover, the TiN/TiO_2_ back surface is shown in Figure 1c, after the deposition of TiN, the diameter is increased to ~330 nm. The cross-section of TiN/TiO_2_ is shown in Figure 1d with a length of about 1.0 µm. The bottoms of the tubes become rough with extremely small nanofeatures after TiN deposition. This increases the effective surface area and surface-active sites, allowing TiN to perform its primary function as a plasmonic layer for light absorption. Figure S2 (Supplementary Data) also shows a top-view SEM image of Au/TiN/TiO_2_/Al_2_O_3_ with agglomerated Au nanoparticles covering the top-surface and template pores with an average diameter of 285 nm.

The structures and chemical constructions of the prepared materials are confirmed using XRD analyses as shown in Figure 2. The sharp peaks in the XRD of the Al_2_O_3_ template, Figure 2a, refer to the formation of a crystalline structure with excellent precision and ordering [32]. The Al_2_O_3_ has three peaks at 44.5°, 65.0°, and 78.1°, which correspond to the growth directions (113), (214), and (119), respectively. In addition, Figure 2a shows the XRD analysis of the synthesized TiN/TiO_2_, which shows eight peaks at 25.7°, 38.0°, 48.2°, 54.2°, 55.2°, 62.7°, 69.7°, and 75.4°. These peaks, coupled with the growth directions (101), (103), (200), (105), (211), (213), (118), and (215), indicated the synthesis of anatase TiO_2_. The deconvolution of the broadband peak in the XRD pattern of TiN/TiO_2_ exhibits the overlapping of two diffraction peaks: (101) peak of anatase-TiO_2_ (JCPDS 21-1272) and the adjacent (111) peak of TiN (JCPDS 38-1420). Otherwise, the deposition of ultra-thin TiN film using DC sputtering on TiO_2_ does not create any phase change in the XRD pattern [33]. From the XRD of TiN/TiO_2_ and the observed peaks for the anatase TIO_2_, it can be concluded that the TiO_2_ layer was crystalline with preferred growth orientation alongside the (101) direction.

The EDX analyses of the Al_2_O_3_ template and TiN/TiO_2_ are shown in Figure 2. For Al_2_O_3_, the O and Al elements of the template are detected. For TiN/TiO_2_ nanotube; Ti, O, and N signals are detected. In addition, Figure S3 (Supplementary Data) shows the EDX spectrum for Au/TiN/TiO_2_/Al_2_O_3_, in which all elements (Ti, O, N, Al, and Au) are well detected. The weight% of the Au is approximately 3%.

### 3.2. Optical Properties of TiN/TiO_2_/Al_2_O_3_

**Figure 3:**
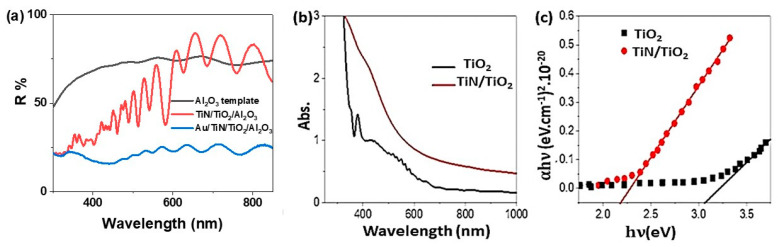
Optical analyses; (**a**) reflectance (Al_2_O_3_ template, TiN/TiO_2_/Al_2_O_3_, and Au/ TiN/TiO_2_/Al_2_O_3_), (**b**) absorption, and (**c**) bandgap calculation for TiO_2_ and TiN/TiO_2_ nanomaterials.

The optical properties of the prepared template and TiN/TiO_2_/Al_2_O_3_ are illustrated in Figure 3. The reflectance spectra of the Al_2_O_3_ template and TiN/TiO_2_/Al_2_O_3_ are displayed in Figure 3a. The reflectance of TiN/TiO_2_/Al_2_O_3_ shows stronger interference fringes than that of the Al_2_O_3_ template alone. In the case of TiN/TiO_2_/Al_2_O_3_, constructive and destructive interferences between reflected waves from TiO_2_/Al_2_O_3_, TiN/TiO_2_, and air/TiN interfaces are responsible for these strong interference fringes. These interferences are low in the UV and Vis regions and increase in the near IR region. The strength of the interference ripples grows with increasing wavelength; nevertheless, the interference ripple below 600 nm is much stronger. This could be due to the Al_2_O_3_ blue emission band, which is attributed to mixed emission from F and F^+^ centers [34]. After Au nanoparticles deposition, the reflectance values decrease, this indicates the increasing absorbance values. This confirms the main plasmonic role of Au nanoparticles. The reduction of the reflectance in the UV/Vis region indicating that the TiN/TiO_2_ materials have strong absorbance in the UV/Vis regions. To confirm that for the TiN/TiO_2_, we have measured the absorbance spectra for TiO_2_ and TiN/TiO_2_ materials, as declared in Figure 3b. Because the anatase bandgap forms between the Ti3d and O2p states, TiO_2_ exhibits a good absorbance [34,35]. After TiN deposition, the absorbance is enhanced in which the right edge of the absorption peak is extended in the visible region till 600 nm. This improvement in the absorption behavior came from the main role of the plasmonic TiN material. The bandgap (Eg) values for TiO_2_ and TiN/TiO_2_ materials, which are 3.1 and 2.2 eV, respectively, demonstrate this improvement in optical properties (Figure 3c). The Eg value was calculated using the Tauc equation, Equation (1), the optical absorption (A), absorption coefficient (α), material thickness (d), light frequency (v), Planck constant (h), and Boltzmann constant (β) [36]:

(1) $$\alpha h\nu \;=\;\beta {\left(h\nu -\mathrm{Eg}\right)}^{\frac{1}{2}}$$

(2) $$\alpha =\left(\frac{2.303}{d}\right)A$$

### 3.3. The Photoelectrochemical Performance

**Figure 4:**
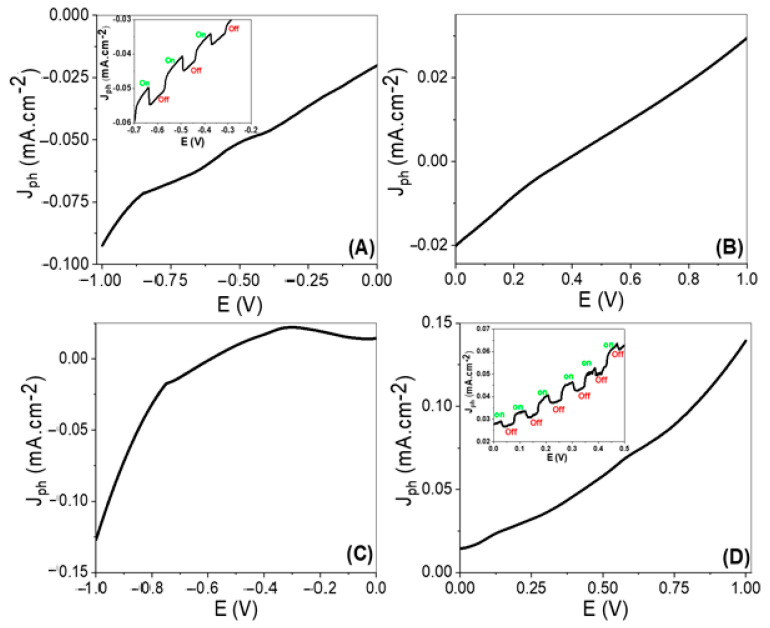
The current density (J_ph_) –voltage curves for (**A**,**B**) TiN/TiO_2_/Al_2_O_3_ and (**C**,**D**) Au/TiN/TiO_2_/Al_2_O_3_ electrodes.

The electrochemical performances of the prepared electrodes, TiN/TiO_2_/Al_2_O_3_ and Au/TiN/TiO_2_/Al_2_O_3_, for the H_2_ generation are measured under lighting from a metal-halide Lamp (Newport, 66926-500HX-R07). The measurements were carried out using the Keithley measurement source unit (model:2400), whereas the J_ph_ value represents the H_2_ generation rate from the waste H_2_O (sewage water). The relation between current density and voltage for the TiN/TiO_2_/Al_2_O_3_ electrode with and without Au coating is revealed in Figure 4A,B shows how the TiN/TiO_2_/Al_2_O_3_ electrode functioned as a photocathode for the generation of H_2_. Under light irradiation, the resultant current density is −0.0924 mA·cm^−2^ @ −1 V, as shown in Figure 4A. Figure 4B shows that this electrode’s performance for O_2_ production is poor, with a current density of just 0.0295 mA·cm^−2^ @ 1 V and an onset value of −0.0202 mA·cm^−2^ @ 0 V. While the Au-coated electrode in Figure 4C,D is utilized to split water and produce O_2_, the opposite electrode produces H_2_ (graphite-electrode). With an onset value of 0.0143 mA·cm^−2^ @ 0 V, the current density was −0.127 mA·cm^−2^ @ −1 V and 0.140 mA·cm^−2^ @ 1 V. As a result, the Au/TiN/TiO_2_/Al_2_O_3_ electrode performed better as a photoanode for O_2_ generation, as shown in Figure 4C,D). The generated current density has increased significantly, Figure 4D, reaching 0.140 mA·cm^−2^. This enhancement in the produced current density is due to the plasmonic resonance of Au nanoparticles and their role in the enhancement of near IR absorbance and hot-electron allocation [36,37,38]. In addition to that, the existence of Au nanoparticles on the electrode surface causes light multiple reflections and mean photon path elongation alongside the catalytic electrode. Additionally, because the Fermi level (EF) of Au NPs is lower than that of TiO_2_, light excites electrons at the Fermi level of plasmonic Au NPs and raises them to the localized surface plasmon energy level. This permits surface plasmon-generated hot electrons to be transported to the conduction band (CB) of TiO_2_, resulting in enhanced charge carriers and photocurrent. Also, Au nanoparticles facilitate the excitation of the electron to overcome the Schottky barrier and reach the conducting band of TiO_2_/TiN. Also, plasmonic Au NPs were able to widen the light absorption band to the visible region. As a result, the incorporation of Au NPs reduces electron-hole recombination and improves light absorption, resulting in improved photocatalytic activity. 

The chopped current density versus the applied voltage is illustrated in Figure 4A,B. From Figure 4A, there is a change in the current density value with on and off light. This indicates the high activity of the TiN/TiO_2_/Al_2_O_3_ electrode for water splitting and the PEC H_2_ generation process. Also, Au/TiN/TiO_2_/Al_2_O_3_ electrode chopped current density is appeared clearly with on and off light.

**Figure 5:**
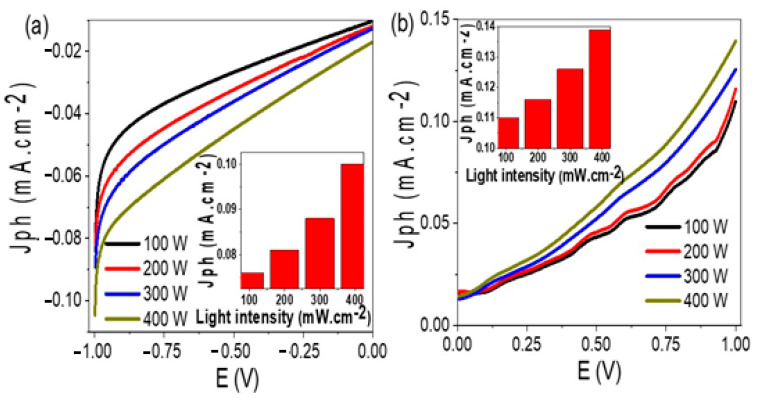
The effect of incident light intensity on the current density-voltage characteristics for (**a**) TiN/TiO_2_/Al_2_O_3_ and (**b**) Au/TiN/TiO_2_/Al_2_O_3_ electrodes.

The effect of light intensity on the prepared TiN/TiO_2_/Al_2_O_3_ electrode is referred to in Figure 5a. The produced J_ph_ increases with increasing the light intensity from 100 to 400 mW. This is clearly appearing in the inset figure, whereas the J_ph_ is increased from 0.076 to 0.096 mA·cm^−2^, respectively. As the light intensity increased, the J_ph_ increases due to the increasing electron-hole pair generation [39,40]. With increasing the light intensity, many photons per second reach the active sites of the photocatalytic material to generate free electrons at the active sites of the TiO_2_/TiN catalyst. The produced current is directly proportional to the absorbed light intensity [41]. The J_ph_ represents the current density produced in the cell due to the water splitting, in which J_ph_ can represent the rate of H_2_ or O_2_ evolution [41,42].

On the other hand, after coating the electrode with Au nanoparticles, the produced J_ph_ value increases, Figure 5b, in which the J_ph_ value changed from 0.10 to 0.139 mA·cm^−2^ with increasing the light intensity from 100 to 400 mW·cm^−2^, respectively. 

The quantum efficiency (QE) is the relation between the incident light photons (intensity) and produced electrons (current intensity), in which QE can be calculated using Equation (4) [43], whereas the photon flux (number of photons per second), N, can be calculated using Equation (3).

(3) N=P·λ/hc

(4) $$\mathrm{QE}=J_{\mathrm{ph}}C/N=J_{\mathrm{ph}}\cdot h\cdot C\cdot c/P\cdot \lambda$$

Here, h is the Plank’s constant (6.626 × 10^−34^ J/s), c is the light speed in space (3 × 10^8^ m/s), P is the light intensity (W·m^−2^), and λ is the light wavelength (405 × 10^−9^ m).

**Figure 6:**
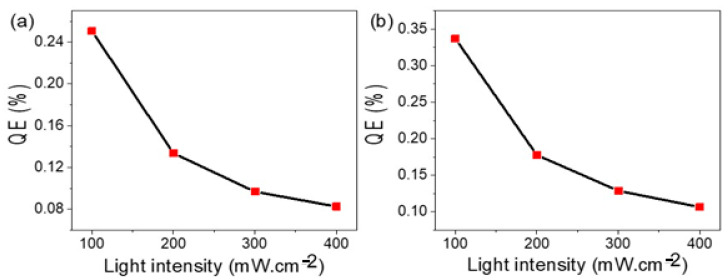
The relation between quantum efficiency and light intensity for (**a**) TiN/TiO_2_/Al_2_O_3_ and (**b**) Au/TiN/TiO_2_/Al_2_O_3_ electrodes at 405 nm.

The value of N is directly proportional with the light intensity as shown in Figure S4, in which N is changed from 2 × 10^21^ to 8 × 10^21^ photon/sec as the light intensity changed from 100 to 400 mW·cm^−2^. On the other hand, the QE and light intensity are almost inversely proportional to each other. The QE for TiN/TiO_2_/Al_2_O_3_ is changed from 0.25% to 0.08% with changing the light intensity from 100 to 400 mW·cm^−2^, Figure 6a. Also, the QE for Au/TiN/TiO_2_/Al_2_O_3_ is changed from 0.34% to 0.1% with changing the light intensity from 100 to 400 mW·cm^−2^, Figure 6b. From Figure 6a,b, it can be seen that there is more enhancement in the QE after Au coating. This is related to the role of Au in enhancing the catalytic properties of the catalyst and improving light absorption. The contact of the electrode with the sewage water causes increasing of the local charges around Au nanoparticles. These local charges enhance the photocatalytic activity of the electrode and increase the water-splitting reaction rate. Furthermore, due to the strong corrosion resistance of Au under most environmental conditions, the layer of the Au nanoparticles on the surface protects the underlying layers and increases the photoelectrode stability [44].

**Figure 7:**
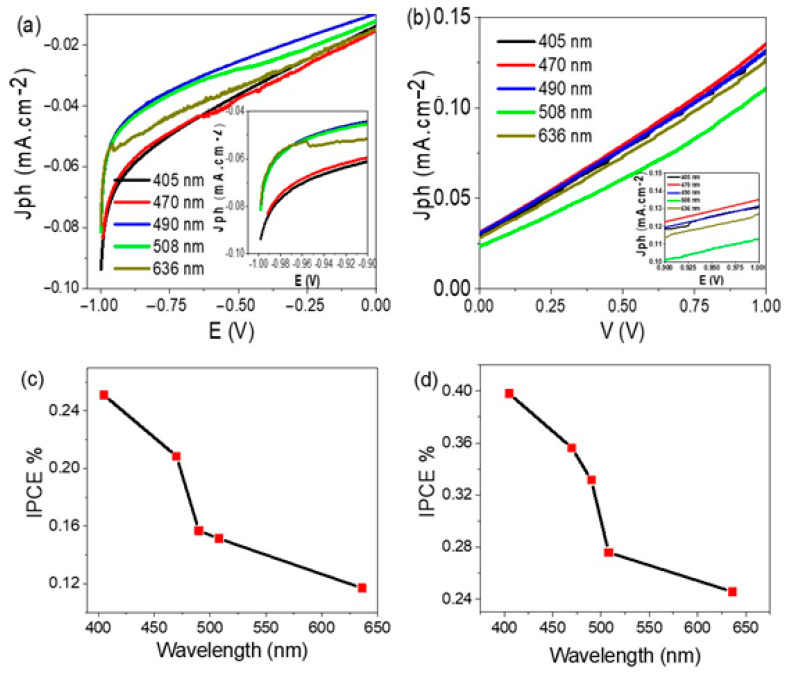
(**a**,**b**) The current density-voltage characteristics under the effect of monochromatic light, and (**c**,**d**) the IPCE for TiN/TiO_2_/Al_2_O_3_ and Au/TiN/TiO_2_/Al_2_O_3_ electrodes, respectively.

The effect of monochromatic light on the produced J_ph_ for TiN/TiO_2_/Al_2_O_3_ and Au/TiN/TiO_2_/Al_2_O_3_ electrodes is shown in Figure 7a,b, respectively. From Figure 7a, it can be seen that the J_ph_ value is changed under the monochromatic light effect, in which 405 nm light has the optimum J_ph_ value (−0.092 mA·cm^−2^). This behavior is matched well with the absorption light spectrophotometry for this electrode.

On the other hand, the effect of monochromatic light on Au/TiN/TiO_2_/Al_2_O_3_ electrode is shown in Figure 7b; the J_ph_ value is changed with the wavelength of the incident light. The irradiance power versus the optical wavelength (irradiance spectrum) of the 66,142–500W Hg (Xe) lamp is presented in Figure S5. The wavelength 470 nm has the optimum J_ph_ value (0.137 mA·cm^−2^), then wavelength 405 nm has a value of 0.131 mA·cm^−2^. The incident photon to current conversion efficiency (IPCE) describes the photocurrent produced due to photon flux. It can be calculated from the wavelength values [45]. The IPCE is calculated from Equation (5).

(5) $$\mathrm{IPCE}=\frac{J_{\mathrm{ph}}\left(\mathrm{mA}\cdot {\mathrm{cm}}^{-2}\right)\cdot 1240\;\left(V\cdot \mathrm{nm}\right)}{P\left(\mathrm{mW}\cdot {\mathrm{cm}}^{-2}\right)\cdot \;\lambda \left(\mathrm{nm}\right)}$$

The IPCE is calculated for Al/Al_2_O_3_/TiO_2_/TiN as shown in Figure 7c at a light intensity of 100 mW·cm^−2^. From Figure 7c, it can be seen that the electrode has the optimum IPCE of 0.25% at 405 nm. This value is decreased by increasing the wavelength to 636 nm. Moreover, after coating the electrode with Au nanoparticles, the IPCE increases to 0.39% at 405 nm. Then, the value decreases with increasing the wavelength from 405 to 636 nm. The IPCE value results from the effect of the photocatalytic property of the electrode for sewage water splitting without adding an external electrolyte. Therefore, the electrode works for converting the sewage water to H_2_ and O_2_ with higher efficiency than that reported by other previous studies [28,46,47,48,49,50].

### 3.4. Effect of Temperature and Thermodynamic Parameters

**Figure 8:**
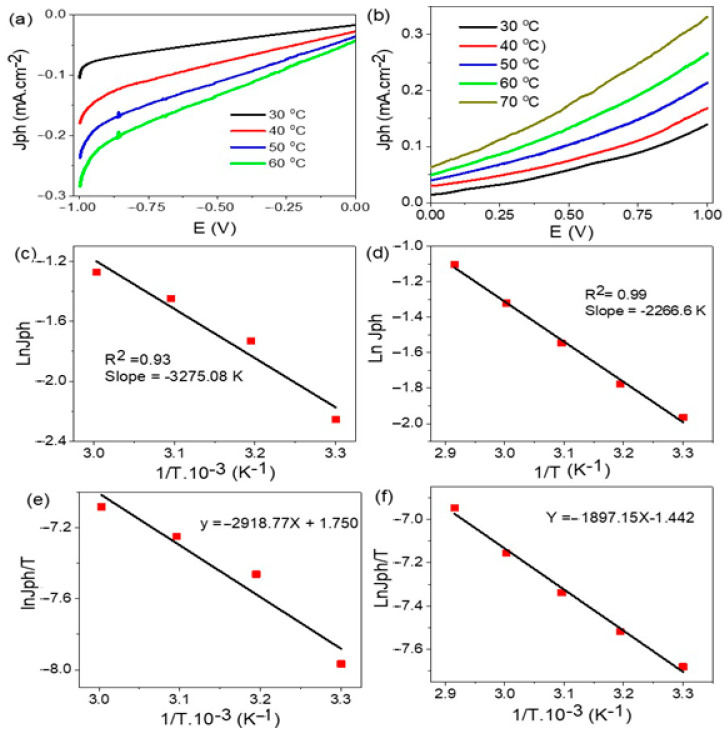
The temperature effect, activation energy, and heat of reaction on (**a**,**c**,**e**) TiN/TiO_2_/Al_2_O_3_ and (**b**,**d**,**f**) Au/TiN/TiO_2_/Al_2_O_3_ electrodes, respectively.

The temperature effect on the TiN/TiO_2_/Al_2_O_3_ electrode for H_2_ production due to water splitting is presented in Figure 8a, in which the J_ph_ increases from −0.1 to −0.28 mA·cm^−2^ with increasing the temperature from 30 to 60 °C. On the other hand, the temperature has the same effect on the electrode after Au coating, Au/TiN/TiO_2_/Al_2_O_3_, as shown in Figure 8b. in which the J_ph_ value increases from 0.13 to 0.33 mA·cm^−2^ with increasing the temperature from 30 to 70 °C, respectively. The general increases in J_ph_ values represent the reaction rate, which reflects the H_2_ and O_2_ production rate [51,52].

From the Arrhenius equation, Equation (6), the activation energy (Ea) can be calculated depending on the particles collision and the rate of water splitting, in which k, R, and A are the rate, universal gas, and Arrhenius constants, respectively, and T is the absolute temperature [53]. From the Ea value, the degree of reaction occurrence is determined [54,55,56].

(6) $$k={\mathrm{Ae}}^{-\mathrm{Ea}/\mathrm{RT}}$$

From Figure 8c,d, it can be seen that the slope values of the relation ln J_ph_ and 1/T give Ea values. The Ea values are 27.22 and 18.84 kJ·mol^−1^ for electrodes Al/Al_2_O_3_/TiO_2_/TiN and Al/Al_2_O_3_/TiO_2_/TiN/Au, respectively. The Ea values for both electrodes are low when compared to earlier published data for other photocatalysts [54,55,56,57,58]. So, the prepared electrodes are efficient for H_2_ and O_2_ evolution due to the water-splitting reaction [57]. Therefore, the Au coating over the Al/Al_2_O_3_/TiO_2_/TiN electrode has a significant role in decreasing the Ea energy and increasing the probability of water-splitting occurrence. This is related to the plasmonic effect of Au nanoparticles and light capture phenomena [58]. In the same manner, the enthalpy (ΔH*) and entropy (ΔS*) can be estimated from the Eyring equation, Equation (7), using the Boltzmann constant (kB) and the Planck constant (h) [59,60].

(7) $$k=T\cdot \frac{\mathrm{kB}}{h}\cdot {\;e}^{\Delta S/R}\cdot e^{-\Delta H/\mathrm{RT}}$$

For calculating the ΔH* and ΔS* values, the slope, and intercept from Figure 8e,f are used. The values of ΔH* for TiN/TiO_2_/Al_2_O_3_ and Au/TiN/TiO_2_/Al_2_O_3_ electrodes are 24.26 and 15.77 J·mol^−1^, respectively, while ΔS* values are 238.1 and 211.5 kJ^−1^·mol^−1^.

### 3.5. Stability of the Photoelectrode

**Figure 9:**
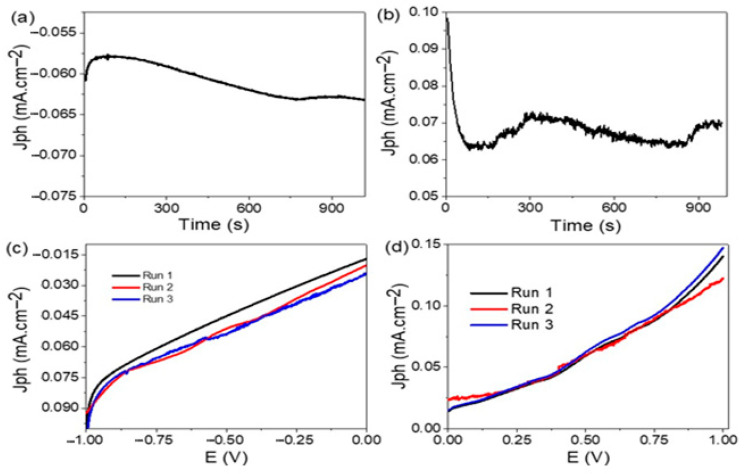
The stability and reproducibility of (**a**,**c**) TiN/TiO_2_/Al_2_O_3_ and (**b**,**d**) Au/TiN/TiO_2_/Al_2_O_3_ electrodes.

The stability of the prepared TiN/TiO_2_/Al_2_O_3_ and Au/TiN/TiO_2_/Al_2_O_3_ photoelectrodes was studied as illustrated in Figure 9a,b. The stability represents the relation between time and the produced J_ph_ value by applying a potential of −0.9 and 0.9 V on the two electrodes before and after Au coating, respectively. From Figure 9a, the electrode without Au is stable with time (1000 s) at about −0.06 mA·cm^−2^. After Au coating, the J_ph_ value becomes stable at about 0.07 mA·cm^−2^, Figure 9b. These values indicate the TiN and Au coating materials have high stability and anticorrosion properties that maintain the produced J_ph_ at the same values with time [13,61]. This means that every small change in the produced J_ph_ value refers to a small corrosion reaction due to the acidity of the sewage water.

As illustrated in Figure S6, the number of H_2_ and O_2_ moles was determined using Equation (8) [62,63], where F is the Faraday constant and dt is the time change.

(8) $$H_{2}\;\mathrm{mole}=\int_{0}^{t}J_{\mathrm{ph}}\cdot \mathrm{dt}/F$$

According to the graph, both gases increase with time, corresponding to 7369.6 and 7915.0 mol h^−1^ cm^−2^ for H_2_ and O_2_, respectively.

The reproducibility of the TiN/TiO_2_/Al_2_O_3_ and Au/TiN/TiO_2_/Al_2_O_3_ photoelectrodes is demonstrated in Figure 9c,d, respectively, by repeating the relation between current density and voltage three times at 30 °C under the simulated sunlight. The standard deviation (SD) values for the two photoelectrodes are 0.3% and 1.29%, respectively.

For comparison, the performance indicators, J_ph_ and IPCE, of the prepared electrodes are displayed in Table 1 relative to some previously reported electrodes for the water-splitting reaction to evaluate O_2_ and H_2_ [28,46,47,50,63,64]. In addition to the low cost and high controllability of the prepared nanomaterials, our prepared photoelectrode has relatively high IPCE and J_ph_ values comparative to the previously reported photoelectrodes in Table 1.

### 3.6. Mechanism of Electron Transition

**Figure 10:**
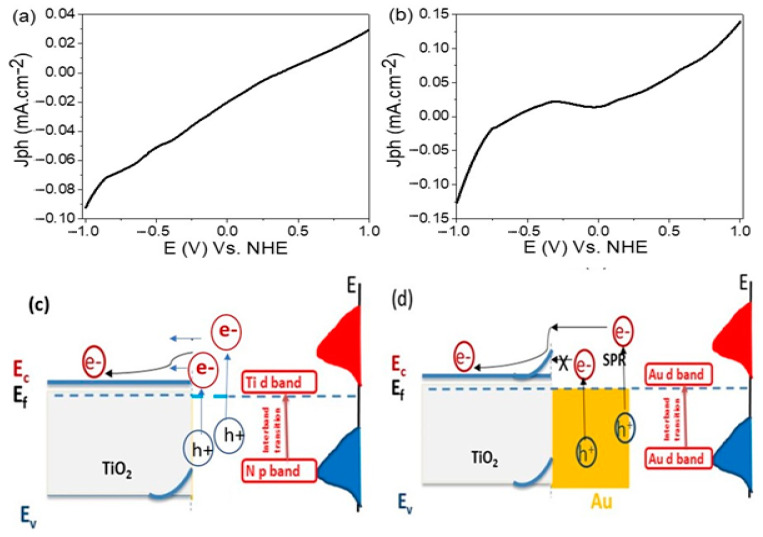
The current density-voltage characteristics and mechanism of electron transition for (**a**,**c**) TiN/TiO_2_ and (**b**,**d**) Au/Tins/TiO_2_.

There are two types of photocatalytic mechanisms for TiN/TiO_2_ and Au/TiN/TiO_2_: (1) photo-induced hot-electron transfer to an adjacent reactant, namely interfacial charge transfer, and (2) localized surface plasmon resonance (LSPR)-based electron-hole separation, namely radiative energy transfer, which requires the plasmonic band to be connected with the TiO2 band gap. Optical and electrochemical experiments were used to investigate the two types (Figure 3a, Figure 4 and Figure 10). Furthermore, the number of H_2_ and O_2_ moles has been computed (Figure S6).

To boost the photocatalytic optical trapping capability and photoelectric conversion rate, this approach uses LSPR to produce enhanced local electromagnetic fields around the TiO_2_ photocatalyst. The aim is to create finely regulated TiN or Au nanostructures with suitable LSPR coupling to the incident light [65,66]. Plasmonic improves light absorption and broadens TiO_2_ absorption in this case [67]. The increased electromagnetic field formed by the coupling between incident light and plasmonic materials transmits radiative energy from the TiN or/and Au plasmonic materials to the semiconducting TiO_2_. The UV-visible spectra of the TiN/TiO_2_ reveal absorption bands between 350 and 600 nm representing the surface plasmon resonance energy, as shown in Figure 3b. The intense absorption is associated with the considerable enhancement in the electromagnetic field in the vicinity of the TiN nanomaterials. As a result, it is realistic to expect TiN nanoparticles to enable a long-lived LSPR process with high-optical cross-sections and tunable throughout a broad energy range, including deep into the UV and Vis [68].

When plasmonic materials’ LSPR modes are coupled with TiO_2_, the resultant broadens, resulting in increased coupling efficiency with incident light. By enhancing light absorption, promoting electron-hole creation, and heating the surrounding area, this enhanced field can be used to improve photocatalytic performance [69]. When a TiO_2_ photocatalytic layer is applied to the plasmonic structure, the increased field is confined to the interior of the TiO_2_ layer, and the plasmon-induced electron-hole pairs diffuse to the photocatalytic surface, contributing to the photocatalytic process. The plasmonic response in the near UV spectrum rises with size t, it can be concluded.

The increased electromagnetic field formed by the interaction of incident light and plasmonic materials transfers radiative energy from the plasmonic materials to the semiconducting TiO_2_ [70]. The radiative energy boosts the photocatalysis efficiency when the LSPR energy is coupled with the TiO_2_ bandgap, otherwise, the energy is more likely to decay nonradiatively through electron–phonon interactions and no significant enhancement in the photocatalytic activity can be observed [71].

At the TiO_2_/plasmonic interface, a significant redistribution of charge occurs depending on the plasmonic work function and the semiconductor electron affinity. In the n-type TiN/TiO_2_ and Au nanomaterials, the charge redistribution forms the Schottky barrier which builds up an internal electric field inside the photocatalyst. The electric field facilitates the transfer of the photoexcited electrons and suppressing electron/ hole recombination and improving the quantum efficiency of photocatalysis.

The TiN nanostructures possess a localized plasmonic resonance in the visible and near IR region, similar to the Au nanostructures [72,73]. The difference between the behavior of TiN and Au nanostructure is that TiN produces Ohmic junction whereas Au forms Schottky junction with TiO_2_ material [64]. Moreover, TiN is a low-cost material with corrosion resistance and mechanical strength properties. The enhancement in the photocatalytic properties with incorporation in the TiO_2_ materials is related to the conversion of energy into electrons below the bandgap of TiO_2_ but limits the potential barrier. TiN has a work function QM of about 4 eV in a vacuum, which matches with the TiO_2_ electron affinity, and this forms an energetic alignment for the hot electrons [74].

From Figure 10a,c, it can be seen that the fabricated TiN/TiO_2_ photoelectrode has an Ohmic junction, which facilitates the electron transition to TiO_2_ without barriers. These electrons contribute to the water-splitting reaction and the negative potential is expected to facilitate the H_2_ production. On the other hand, incorporating Au nanoparticles boosts photocurrent density while simultaneously forming a Schottky circuit, as seen in Figure 10b,d [74]. As a result, not all electrons absorbed by Au nanoparticles can travel to TiN and TiO_2_ materials for water splitting (cold electrons), while hot electrons can only pass through the barrier for the water-splitting reaction. So, the addition of Au nanomaterial increases the potential to be a positive value.

## 4. Conclusions

This work is very interesting for H_2_ production from sewage water using TiN/TiO_2_/Al_2_O_3_ nanotube material. The Al_2_O_3_ template has been prepared with high accuracy and ordering with hexagonal pores using the Ni imprinting method. TiN/TiO_2_ is deposited inside the pore using ALD and DC sputter-coating method, respectively. Characteristic analyses of the structural, morphological, and optical properties were carried out for the prepared template and nanotube materials. The application of the TiN/TiO_2_/Al_2_O_3_ materials as photocathode carried out with and without Au nanoparticles coating, in which the Au materials facilitate the sewage water-splitting reaction. The effect of different parameters was studied for water-splitting reaction, such as light intensity, light wavelength, and temperature. The values of QE and IPCE were calculated with a value of 0.34 and 0.39% at 400 nm. Moreover, all thermodynamic parameters are calculated for the photocathode for H_2_ production, wherein Ea, ΔH*, and ΔS* values were 18.84 kJ·mol^−1^, 15.77 J·mol^−1^, and 211.5 kJ^−1^·mol^−1^ for Au/TiN/TiO_2_/Al_2_O_3_ photoelectrode.

## Figures and Tables

**Table 1:** Comparison of the IPCE and J_ph_ values of the prepared photoelectrodes with previous work.

Photoelectrode	Electrolyte	J_ph_	IPCE % (405 nm)	Ref.
BiFeO_3_	H_2_SO_4_	0.1 mA·cm^−2^	0.21	[28]
PrFeO	H_2_SO_4_	−130 µA·cm^−2^	-	[46]
Au/Pb(Zr, Ti)O_3_	H_2_SO_4_	0.06 mA·cm^−2^	0.2	[47]
Poly(3-aminobenzoic acid) frame	H_2_SO_4_	0.08 mA·cm^−2^	-	[50]
TiN-TiO_2_	H_2_SO_4_	3.0 nA·cm^−2^	0.03	[64]
TiN/TiO_2_/Al_2_O_3_ Au/TiN/TiO_2_/Al_2_O_3_	Sewage water Sewage water	0.0.9 mA·cm^−2^ 0.14 mA·cm^−2^	0.25 0.39	Present work

## Data Availability

Not applicable.

## Supplementary Materials

The following are available online at https://www.mdpi.com/article/10.3390/nano11102617/s1, Figure S1: Testing the prepared materials as a photocathodem, Figure S2. SEM image of Au/TiN/TiO_2_/Al_2_O_3,_ Figure S3. EDX of Au/TiN/TiO_2_/Al_2_O_3,_ Figure S4. The relation between the number of photons per second and the incident light intensity, Figure S5. Irradiance spectrum of 66142 500 W Hg(Xe) lamp, Figure S6. The number of moles evolved (a) H_2_ and (b) O_2_ gas, Table S1. The chemical composition of the sewerage water used for the H_2_ generation.
