# Supplementary material for: Nanoporous TiN/TiO2/Alumina Membrane for Photoelectrochemical Hydrogen Production from Sewage Water
Source: Nanomaterials (Basel). 2021 Oct 6;11(10):2617. doi: 10.3390/nano11102617 (PMC8540468; doi:10.3390/nano11102617)
Supplement: Supplementary file 1 [file nanomaterials-11-02617-s001.zip › nanomaterials-1354748-supplementary.pdf]

nanomaterials

Supplementary Data

Nanoporous TiN/TiO_2/Alumina Membrane for Photoelectrochemical Hydrogen Production from Sewage Water

Abdullah Almohammedi [1], Mohamed Shaban [1,*], Huda Mostafa [2], and Mohamed Rabia [2,3]

[1] Department of Physics, Faculty of Science, Islamic University in Madinah, Al-Madinah Al-Munawarah, 42351, Saudi Arabia; ard.almohammedi@hotmail.com
[2] Nanophotonics and Applications (NPA) Lab, Physics Department, Faculty of Science, Beni-Suef University, Beni-Suef, 62514, Egypt; hudamostafa55@gmail.com (H.M.); mohamedchem@science.bsu.edu.eg (M.R.)
[3] Polymer Research Laboratory, Chemistry Department, Faculty of Science, Beni-Suef University, Beni-Suef 62511, Egypt
* Correspondence: mssfadel@aucegypt.edu

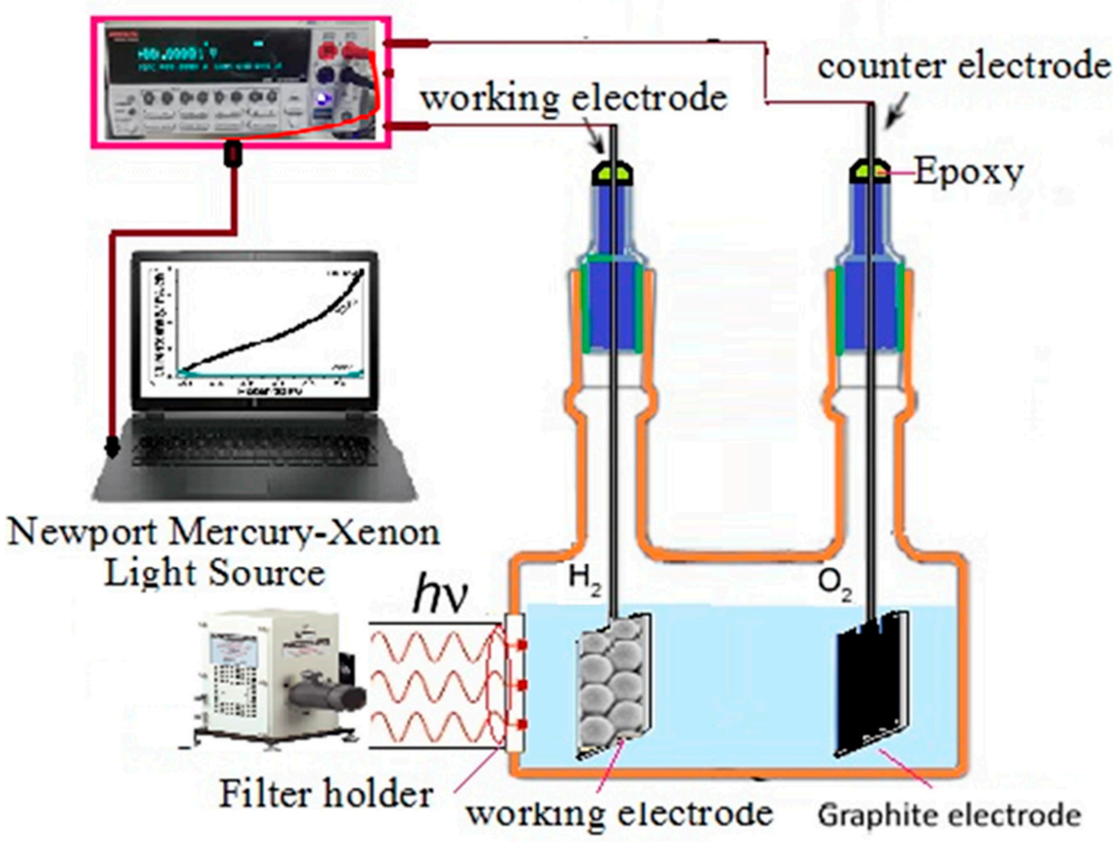

Figure S1. Testing the prepared materials as a photocathode.

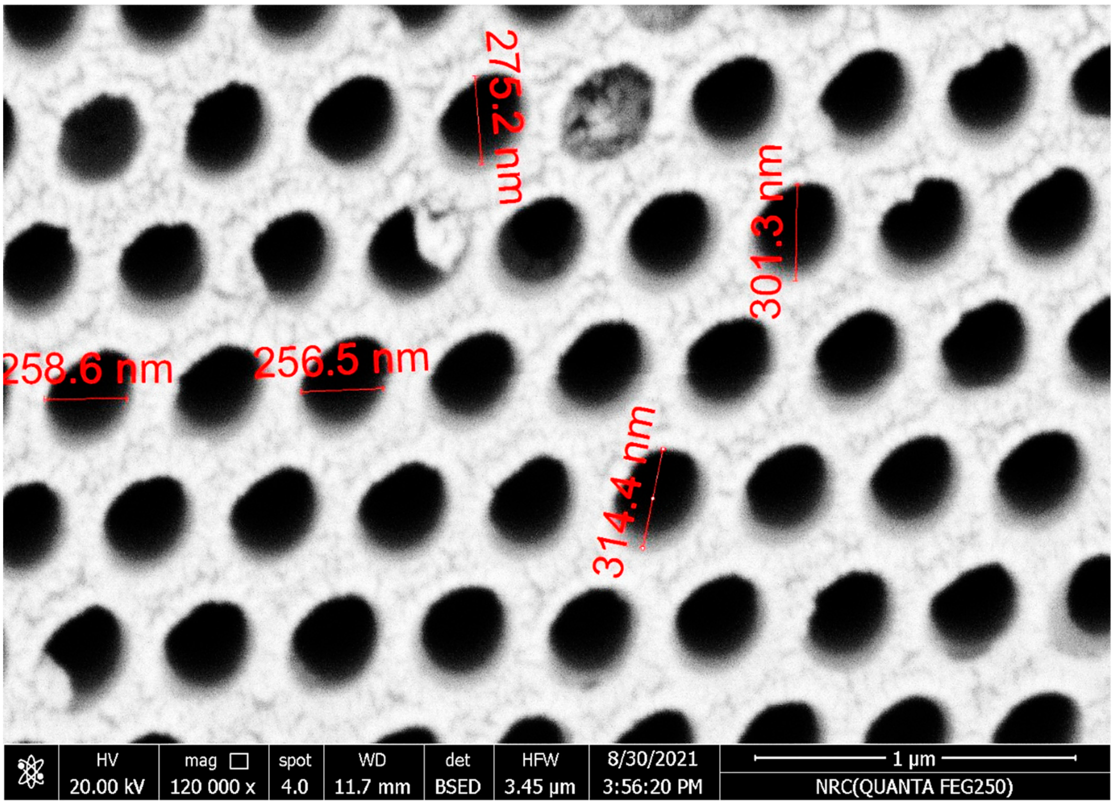

Figure S2. SEM image of Au/TiN/TiO_2/Al_2O_3.

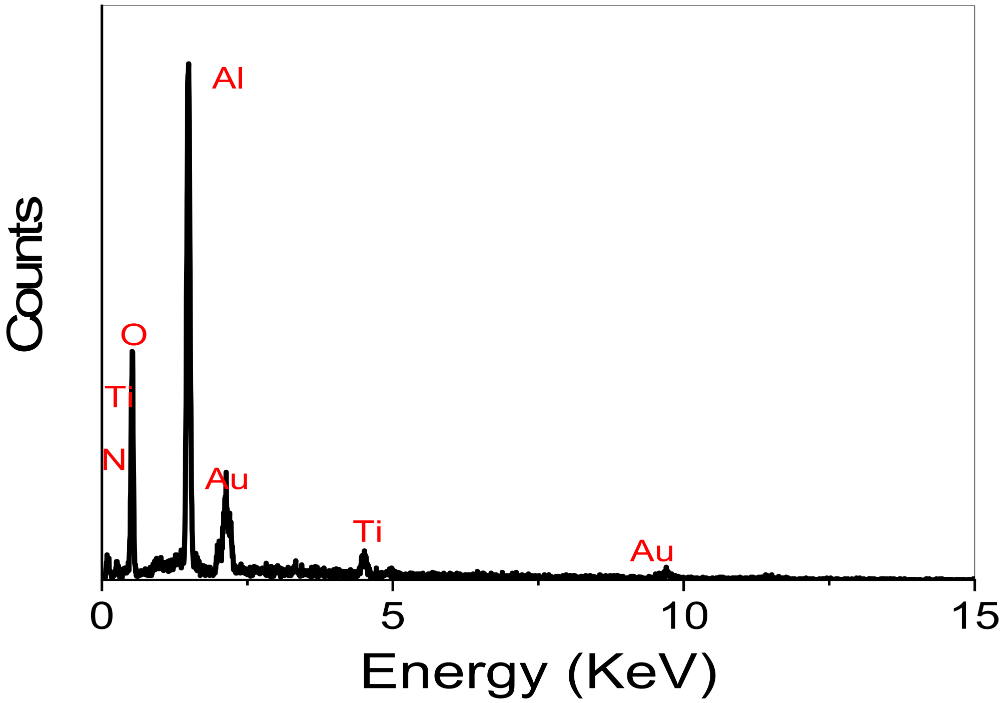

Figure S3. EDX of Au/TiN/TiO_2/Al_2O_3.

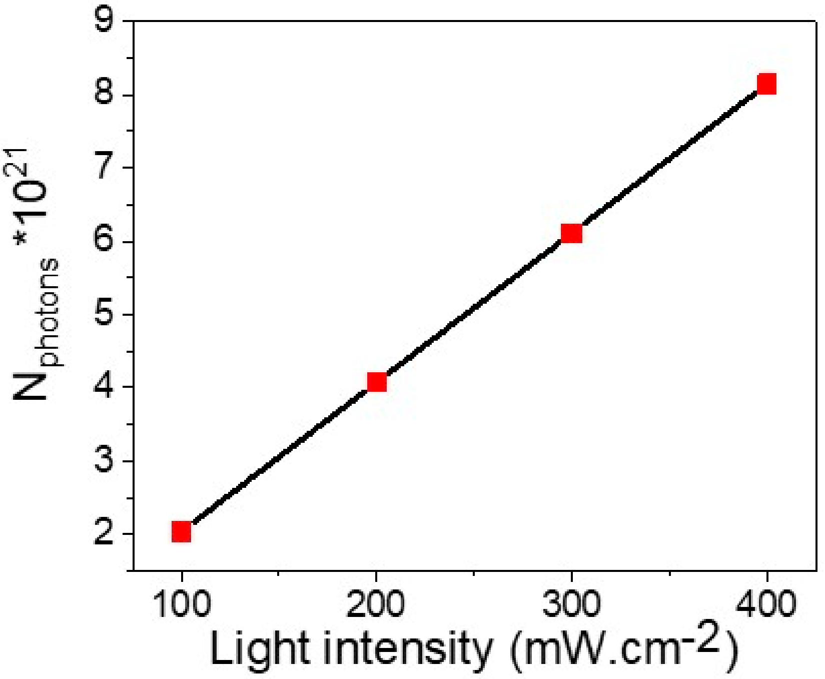

Figure S4. The relation between the number of photons per second and the incident light intensity.

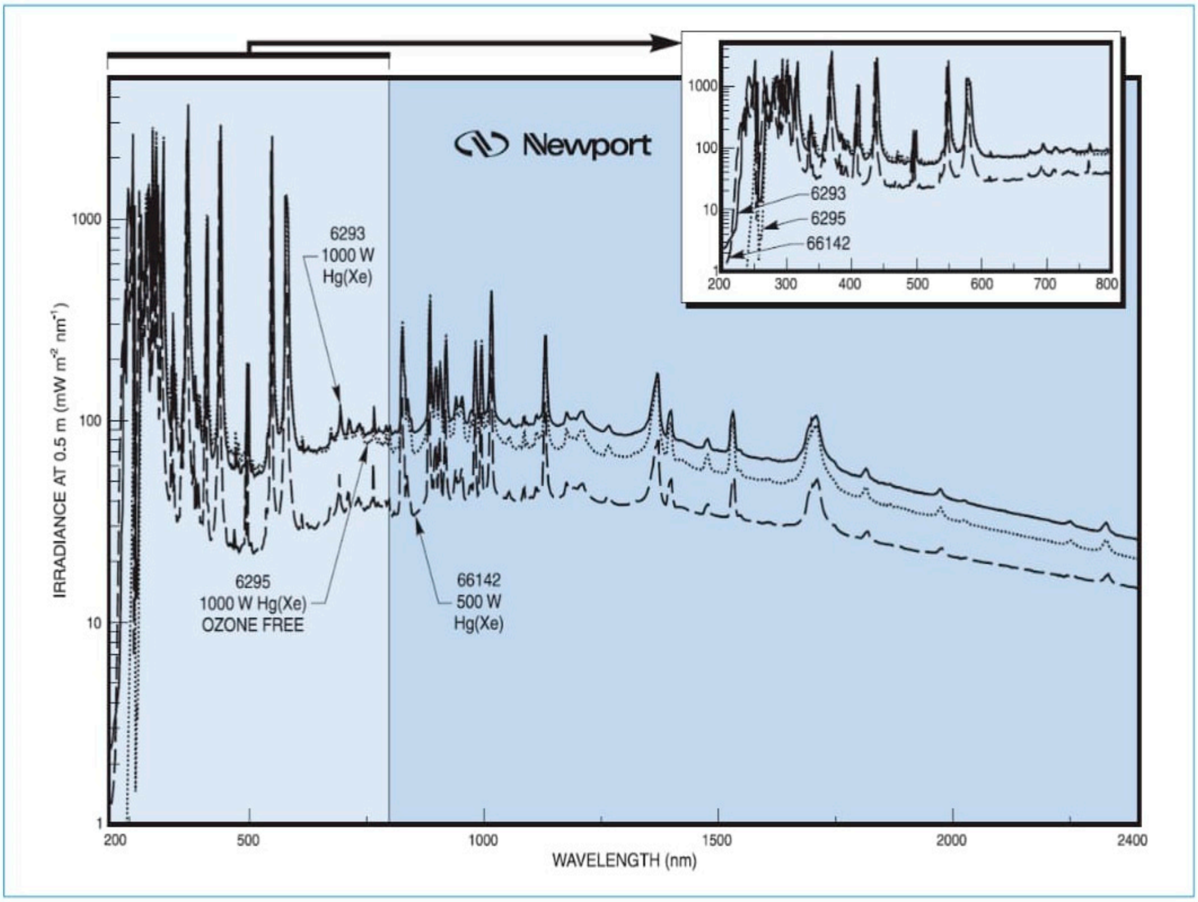

Figure S5. Irradiance spectrum of 66142 500 W Hg(Xe) lamp.

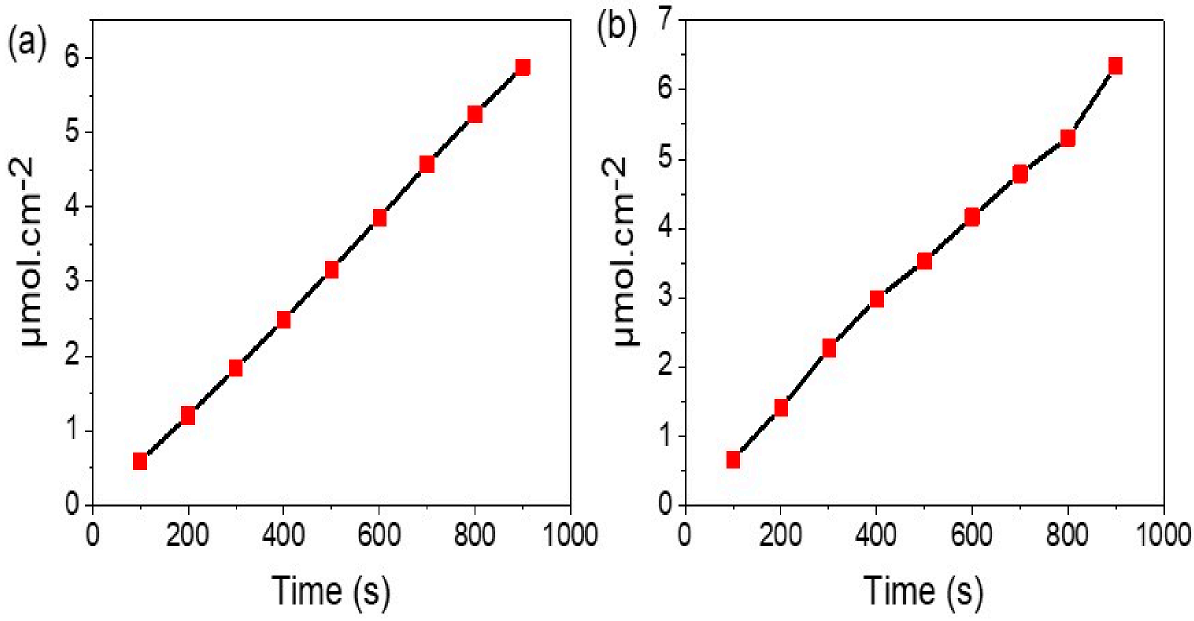

Figure S6. The number of moles evolved (**a**) H_2 and (**b**) O_2 gas.

Table S1. The chemical composition of the sewerage water used for the H_2 generation.

**Material or element**	**Concentration (mg/L)**
Phenols	0.015
F^-	1.0
Al^{3+}	3.0
NH_3	5.0
Hg^{2+}	0.005
Pb^{2+}	0.5
Cd3$^+$	0.05
As^{3+}	0.05
Cr^{3+}	1.0
Cu^{2+}	1.5
Ni^{3+}	0.1
Fe^{3+}	1.5
Mn^{2+}	1.0
Zn^{2+}	5.0
Ag^+	0.1
Ba^{3+}	2.0
Co^{2+}	2.0
Other cations	0.1
Pescides	0.2
CN^{-1}	0.1
Industrial washing	0.5
Coli groups	4000/100 cm^3

Determination of ΔH* and ΔS* values

In the same manner, the enthalpy (ΔH*) and entropy (ΔS*) can be estimated from the Eyring equation, Equations.S1 and S2, using the Boltzmann constant (kB) and Planck constant (h).

$$k = T.\frac{kB}{h}.e^{\Delta S/R}.e^{-\Delta H/RT} \tag{S1}$$

$$Ln(\frac{K}{T}) = Ln\frac{kB}{h} + \frac{\Delta S}{R} - \frac{\Delta H}{RT} \tag{S2}$$

For calculation ΔH*, we have to plot the relation between Ln (K/T) and 1/T, from the slope value, we can determine the ΔH* value. From the same relation, ΔS* value can be determined from the intercept, in which the intercept value equal $Ln\frac{kB}{h} + \frac{\Delta S}{R}$.

For calculating the ΔH* and ΔS* values, the slope, and intercept from Figure 8 (e, f) are used. The values of ΔH* for $TiN/TiO_2/Al_2O_3$ and $Au/TiN/TiO_2/Al_2O_3$ electrodes are 24.26 and 15.77 J.mol-1, respectively, while ΔS* values are 238.1 and 211.5 $JK^{-1}mol^{-1}$.
